# Non-viral expression of chimeric antigen receptors with multiplex gene editing in primary T cells

**DOI:** 10.3389/fbioe.2024.1379900

**Published:** 2024-05-31

**Authors:** Dan Cappabianca, Jingling Li, Yueting Zheng, Cac Tran, Kassandra Kasparek, Pedro Mendez, Ricky Thu, Travis Maures, Christian M. Capitini, Robert Deans, Krishanu Saha

**Affiliations:** ^1^ Department of Biomedical Engineering, University of Wisconsin-Madison, Madison, WI, United States; ^2^ Synthego Corporation, Redwood City, CA, United States; ^3^ Department of Pediatrics, University of Wisconsin-Madison, Madison, WI, United States; ^4^ Carbone Cancer Center, University of Wisconsin-Madison, Madison, WI, United States

**Keywords:** multiplex gene editing, CRISPR/Cas9, GD2, chimeric antigen receptor T cells, PD-1, neuroblastoma, chromosomal translocation

## Abstract

Efficient engineering of T cells to express exogenous tumor-targeting receptors such as chimeric antigen receptors (CARs) or T-cell receptors (TCRs) is a key requirement of effective adoptive cell therapy for cancer. Genome editing technologies, such as CRISPR/Cas9, can further alter the functional characteristics of therapeutic T cells through the knockout of genes of interest while knocking in synthetic receptors that can recognize cancer cells. Performing multiple rounds of gene transfer with precise genome editing, termed multiplexing, remains a key challenge, especially for non-viral delivery platforms. Here, we demonstrate the efficient production of primary human T cells incorporating the knockout of three clinically relevant genes (*B2M*, *TRAC*, and *PD1*) along with the non-viral transfection of a CAR targeting disialoganglioside GD2. Multiplexed knockout results in high on-target deletion for all three genes, with low off-target editing and chromosome alterations. Incorporating non-viral delivery to knock in a GD2-CAR resulted in a TRAC-B2M-PD1-deficient GD2 CAR T-cell product with a central memory cell phenotype and high cytotoxicity against GD2-expressing neuroblastoma target cells. Multiplexed gene-editing with non-viral delivery by CRISPR/Cas9 is feasible and safe, with a high potential for rapid and efficient manufacturing of highly potent allogeneic CAR T-cell products.

## Introduction

Chimeric antigen receptor (CAR) T cells utilize an engineered receptor consisting of a single-chain variable fragment (scFV) specific for an extracellular tumor antigen attached to intracellular signaling domains that can elicit a T-cell response against tumors in an antigen-specific manner independent of human leukocyte antigen (HLA) (June et al., 2018; Hucks and Rheingold, 2019). To date, there are six FDA-approved CAR T-cell therapies for hematologic malignancies such as B-cell acute lymphoblastic leukemia, multiple myeloma, and non-Hodgkin B-cell lymphomas (Chen et al., 2023). However, CAR T-cell therapies have been limited to autologous products and continue to have limited activity against solid tumors, in part due to a lack of homing to the tumor, limited persistence, and engagement of inhibitory signals from the tumor microenvironment that, combined with chronic antigen stimulation, can induce exhaustion (McLane et al., 2019; Kankeu Fonkoua et al., 2022). For example, in a phase-I trial using a third-generation anti-GD2 CAR-T therapy targeting pediatric and young adult osteosarcoma and neuroblastoma, *in vivo* T-cell expansion and persistence were hampered by a lack of memory phenotypes and rampant exhaustion (Kaczanowska et al., 2023). Engineering solutions to resist exhaustion and promote memory formation in pre-infusion CAR T-cell products are urgently needed to more effectively treat solid tumors.

Strategies that prevent exhaustion are one way to improve CAR T-cell function. Cancers often express inhibitory ligands that engage with surface receptors on T cells (PD-1, LAG3, CTLA-4, etc.) that contribute to exhausted phenotypes (Park et al., 2016; He and Xu, 2020). Blocking these receptors with therapeutic antibodies has led to the development of immune checkpoint inhibitor (ICI) therapy (Sharma and Allison, 2015; Sharma et al., 2021). Combining ICI with adoptive T-cell therapies to treat cancer has been shown to increase persistence and effector function, especially with anti-PD-1 (Burga et al., 2015; Gargett et al., 2016; Najafi and Mortezaee, 2023). Alternatively, the CRISPR/Cas9-mediated knockout of inhibitory checkpoint genes to prevent their expression (Jinek et al., 2012) has been used to target PD-1 expression in CAR T cells, and this approach can increase resistance to exhaustion *in vitro* (Rupp et al., 2017; Guo et al., 2018; Hu B. et al., 2019; Hu W. et al., 2019; Choi et al., 2019; Dai et al., 2019; McGowan et al., 2020), with similar results *in vivo* (Lin et al., 2019; Wang et al., 2021; Khan and Sarkar, 2022).

Another approach to minimizing exhaustion is using allogeneic donors to generate an off-the-shelf CAR T-cell product and avoid the lengthy vein-to-vein time characteristic of autologous CAR T-cell therapy. Random integration from viral vectors presents a safety concern for regulatory agencies, which non-viral gene integration in CAR T cells can rectify (Foy et al., 2022; Kath et al., 2022; Madison et al., 2022; Ye et al., 2022; Webber et al., 2023). Virus-free strategies utilizing homology-directed repair of double-strand DNA breaks from CRISPR/Cas9 cleavage can incorporate linearized dsDNA templates into a precise locus. This approach can place CAR transgenes under the control of endogenous promoters, such as the *TRAC* locus (Eyquem et al., 2017; Stadtmauer et al., 2020; Mueller et al., 2022), and yield more controlled transgene expression, copy numbers in the genome (1 or 2), limited off-target effects, and higher fractions of stem-cell memory phenotypes, which correlates with increased T-cell retention *in vivo* (Ren et al., 2017; Nakazawa et al., 2020). An anti-GD2 CD28-OX40 *TRAC-*CAR T-cell product electroporated with a linear dsDNA construct generated by PCR has shown promise in a GD2^+^ human neuroblastoma xenograft model (Sasu et al., 2023). CRISPR/Cas9 has also been used to disrupt the *TRAC* and *B2M* genes to generate ‘universal’ allogeneic CAR T cells that knock out the endogenous TCR and HLA class-1 molecules, respectively (Kebriaei et al., 2016; Magnani et al., 2020), thereby limiting graft-versus-host-disease (GVHD) and immune rejection by T cells in patients. However, the use of CRISPR/Cas9, especially when targeting the *TRAC* locus, can cause chromosomal translocations and off-target effects that must be mitigated to ensure patient safety (Bishop et al., 2021).

In this study, we generated CAR T cells using CRISPR/Cas9-mediated insertion of GD2-CAR transgene at the *TRAC* locus along with simultaneous disruption of the *TRAC*, *β2M*, and *PDCD1* loci with the goal of minimizing GVHD, T-cell rejection, and CAR exhaustion. Triple-knockout GD2-CAR T cells contained a high proportion of naïve and central memory cells in the pre-infusion product, were potent against GD2^+^ human neuroblastoma cells *in vitro,* and highly expressed the CAR receptor while maintaining low levels of translocations and off-target edits. These results demonstrate the feasibility of generating multiplexed edited T cells, which are particularly attractive for generating allogeneic CAR T-cell products.

## Methods

### T-cell isolation

Human primary CD4^+^ and CD8^+^ T cells were isolated from commercially available leukopaks (BioIVT, Westbury, NY) via positive selection on a CliniMACS (Miltenyi Biotec, Auburn, CA) following the manufacturer’s instructions. After obtaining isolated CD4^+^ and CD8^+^ T cells, cell identity was confirmed via flow cytometry.

### T-cell culture

Primary T cells were cultured in RPMI, supplemented with 10% fetal bovine serum (FBS), and activated with anti-CD3/28 Dynabeads (Thermo Fisher Scientific, Waltham, MA), which were used to stimulate T-cell activation for 48–72 h. The media were supplemented with IL-2 (PeproTech, Cranbury, NJ) at 200 U/mL (during activation) or 500 U/mL (during expansion), IL-15 (PeproTech) at 5 ng/mL, or IL-7 (PeproTech) at 5 ng/mL. Cells were counted and passaged every 2 days to a density of one million cells/mL.

### Plasmid constructs

GD2-tNGFR-CAR: the GD2-OX40-CD28-CD3ζ CAR (∼1.6 kb) sequence was a gift from Malcolm Brenner (Baylor College of Medicine) and modified for insertion by CRISPR/Cas9, as published previously (Sasu et al., 2023), but with an additional truncated nerve growth factor receptor (tNGFR) (∼0.8 kb) tag. All plasmids were expanded and purified *via* Midiprep (Azenta, Chelmsford, MA). The plasmid sequences can be found in Supplementary Table S1.

### Double-stranded DNA HDR template production

Primers were designed for PCR donor templates from plasmids. Amplicons were generated using NEBNext High-Fidelity PCR Master Mix (NEB, Ipswich, MA). To improve knock-in efficiency, truncated Cas9 target sequences (tCTS) were added at each end of the HDR GD2-donor template. Primers used to amplify GD2-CAR donors can be found in Supplementary Table S2. The thermocycler program consisted of 1) 98°C for 30 s; 2) 98°C for 10 s; 3) 67°C for 30 s; 4) 72°C for 2 min; and 5) 72°C for 5 min, with the repetition of steps 2 to 4 for 40 cycles. PCR products were pooled to conduct solid-phase reversible immobilization (SPRI) cleanup (0.5X) using AMPure XP beads according to the manufacturer’s instructions (Beckman Coulter, Brea, CA). Every 1,000 µL PCR amplicon was mixed with 500 µL AMPure beads. After 5 min of incubation at room temperature, separation and ethanol wash were subsequently followed. DNA elution was conducted at 37°C for 15 min to increase the yield. Amplicons from the first round of clean-up were pooled and subjected to a second round of SPRI cleanup (0.5X), as described above. The dsDNA template concentration was quantified by qubit fluorometric quantification (Thermo Fisher Scientific, Waltham, MA). NanoDrop 2000 was also employed to verify the template purity. All dsDNA subjected to non-viral knock-in experiments was diluted to 2 μg/μL.

### Multiplex editing and non-viral knock-in using primary T cells

Human primary CD4^+^ and CD8^+^ T cells were activated for 48–72 h prior to nucleofection. T cells were debeaded according to the manufacturer’s protocol and counted by Trypan blue exclusion on a NucleoCounter NC-200 (ChemoMetec, Denmark). Prior to nucleofection, ribonucleoprotein (RNP) mixtures with spCas9 (Aldevron, Fargo, ND), single-guide (sg) RNAs (Synthego, Redwood City, CA), and poly-L-glutamic acid (PGA, Sigma-Aldrich, St. Louis, MI) (100 mg/mL) at volumetric ratios of gRNA (1): PGA (0.8): Cas9 (1) were used. To prepare RNP, 10 mg/mL Cas9 (stock concentration: 62 μM) was diluted in Cas9 storage buffer (Aldevron) to 40 μM and then mixed with 40 pmol of spCas9 (1 μL/1e6 cells), 250 pmol of *TRAC* sgRNA (0.375 μL/1e6 cells), *B2M* sgRNA (1 μL/1e6 cells) and *PDCD1* sgRNA (1.13 μL/1e6 cells), and PGA (2 μL/1e6 cells). RNP was then incubated at 37°C for 15–30 min. For CAR T-cell production, 1–2 µg of the dsDNA HDR template was added to the RNP for 5 min at room temperature. T cells were centrifuged at 400 *g* for 5 min, re-suspended in 18 uL of the Lonza P3 buffer, and added to the RNP/DNA mixture. T cells were nucleofected using a Lonza 4D-Nucleofector (Lonza, Walkersville, MD), with programs EO-115 for multiplex knockout and EH-115 for non-viral CAR knock-in. Edited T cells were recovered at 37°C and 5% CO_2_ for 5–10 min in cuvettes with 80 uL of RPMI media supplemented with IL-7 and IL-15 or IL-2. Cells were then added to a 48-well plate and expanded in RPMI media supplemented with IL-7 and IL-15 or IL-2 for 7 days. Guide RNA sequences can be found in Supplementary Table S3.

### T-cell cryopreservation and thawing

T cells were harvested on day 7 post-nucleofection by centrifugation at 400 *g* × 5 min and counted via Trypan blue exclusion. Cells were then re-suspended in CryoStor (STEMCELL Technologies, Cambridge, MA) at 10 million cells/mL and aliquoted into cryovials. For thawing, cells were re-suspended at 2.5 million/mL in the ImmunoCult XF Medium (STEMCELL Technologies) supplemented with 50 U/mL IL-2 (PeproTech) for 24 h.

### Flow cytometry analysis

CAR expression was verified using the 1A7 anti-14G2a antibody (National Cancer Institute, Biological Resources Branch) conjugated to APC using a Lightning Link APC Antibody Labeling Kit (Novus Biologicals, Centennial, CO). TCR expression was detected using an anti-human TCR α/β antibody conjugate to BV421 (BioLegend, San Diego, CA). Beta 2 Microglobulin was detected using an anti-human β2M antibody conjugated to PE (BD, Franklin Lakes, NJ). PD-1 was detected using an anti-human PD-1 (CD279) antibody conjugated to either BV421 or BV510 (BioLegend). CD45 was detected using an anti-human CD45 antibody bound to Spark Blue 574 (BioLegend), CD45RA was detected with an anti-human CD45RA antibody bound to PE-Fire 700 (BioLegend), and CCR7 was detected with an anti-human CCR7 antibody bound to Spark NIR 685 (BioLegend).

Flow cytometry was performed to assess CAR and TCR positivity on day 8 of manufacturing on an Attune NxT flow cytometer (Thermo Fisher Scientific). Immunophenotyping of cells was performed on day 10 of manufacturing using a spectral immunophenotyping panel on an Aurora spectral cytometer (Cytek, Fremont, CA), and fluorescence-activated cell sorting (FACS) was performed on a FACSAria (BD). In brief, cells were plated in a 96-round bottom well plate (1e5 for CAR/TCR and 2.5e5 for spectral immunophenotyping), washed with 200 μL of phosphate-buffered saline (PBS, Gibco), and spun at 1,200 g × 1 min, twice. Cells were then stained for viability with either GhostRed 780 (Cytek) or Live-Dead Blue (Thermo Fisher Scientific). For CAR/TCR staining, 1 μL of GhostRed 780 was added to 10 mL of PBS to make a stock solution, and 100 μL of stock solution was added to each sample and incubated for 30 min in the dark. For spectral flow staining, Live-Dead Blue stain was re-suspended in 50 μL of DMSO, with 1 μL added per 1 mL PBS to make a stock solution, and 200 μL of the stock solution was added to each sample and incubated for 30 min in the dark. After viability dyes were added, samples were washed twice and blocked for 30 min with 50 μL of the FACS buffer (0.5% bovine serum albumin in PBS) with TruStain FcX solution (0.5 μL/sample, Biolegend, San Diego, CA). Antibodies were then added to 100 μL of the BD Brilliant Stain Buffer (Cat # 659611, BD Biosciences, Franklin Lakes, NJ) at the optimized amounts (Supplementary Table S4) and incubated for 1 h. Cells were then washed, re-suspended in 200 or 75 μL of the FACS buffer, and analyzed on the Attune or Aurora, respectively. For spectral immunophenotyping, cells were gated by relative size, shape, singlets, viability, TCR negativity, and CAR transgene positivity to find an analyzable population of viable CAR T cells. All antibodies are listed in Supplementary Table S4.

Analysis of spectral flow cytometry data was performed using Cytek’s SpectroFlo program. Single-positive controls for each color were collected and analyzed in SpectroFlo for positive and negative populations. SpectroFlo’s unmixing algorithm was then used to compensate for spillover and the autofluorescence of cells. Data were then exported to FlowJo, where samples were gated for non-debris, singlets, and live cells. CD45, TCR, and CAR positivity were used to gate cell populations for *in vitro* samples. Representative plots and population percentages were generated in FlowJo using fluorescence minus one control to set positive gates.

### Cell lines

GD2^+^ human neuroblastoma CHLA-20 cells were gifted by Dr. Mario Otto (University of Wisconsin-Madison). These cells were cultured in DMEM supplemented with 10% FBS (Gibco) and 1% penicillin–streptomycin (P/S) (Gibco). AkaLucGFP CHLA-20 cells were created through viral transduction by Dr. James Thomson (Morgridge Institute for Research). Cell authentication was performed using short tandem repeat analysis (IDEXX BioAnalytics, Westbrook, Maine, United States) and per ATCC guidelines using cell morphology, growth curves, and *mycoplasma* testing within 6 months using the MycoStrip *Mycoplasma* Detection Kit (Invitrogen, Waltham, MA). CHLA-20 was maintained in culture at 37°C in 5% CO_2._

### *In vitro* cytotoxicity assay

To assess CAR T-cell potency, AkaLUC-GFP CHLA-20 cells (a gift from Jue Zhang, University of Wisconsin-Madison) were seeded in triplicate on 96-well plates and incubated for 24 h at 37°C. Then, cryopreserved CAR T cells from day 10 of manufacturing were thawed and added to each well at various effector:target ratios. The plate was centrifuged for 5 min at 100 g and then placed in an IncuCyte S3 Live-Cell Analysis System (Sartorius, Gottingen, Germany) and stored at 37°C with 5% CO_2_. Images were taken every 3 h for 48 h. A green fluorescence object count was used to calculate the number of cancer cells in each well, and fluorescent images were analyzed using IncuCyte Base Analysis software.

### ddPCR

Digital droplet (dd)PCR assays were designed for quantifying balanced translocations between *TRAC*, *B2M*, or *PDCD1*, as previously described (Glaser et al., 2023). Readout was performed with QX 100 Droplet Reade (Bio-Rad, Hercules, CA) and ddPCR Droplet Reader Oil (Bio-Rad). Data analysis was conducted using QuantaSoft 1.7.4 (Bio-Rad). Primers and probes were from IDT (Coralville, IA).

### GUIDE-seq

GUIDE-seq experiments were performed as described previously for U2OS (Tsai et al., 2015). In brief, the blunt-ended dsODN used in our GUIDE-seq experiments was prepared by annealing two modified oligonucleotides of the following compositions: 1) ssODN_Sense_str:/5Phos/G*C*TCGCGTTTAATTGAGTTGTCATATGTTAATAACGGTATACGC*G*A and 2) ssODN_Antis_str:/5Phos/T*C*GCGTATACCGTTATTAACATATGACAACTCAATTAAACGCGA*G*C, where Phos represents a 5′ phosphorylation and * indicates a phosphorothioate linkage. 1E6-activated T cells were electroporated (program ER100) with Cas9:sgRNA (40 pmol:100 mol) and 50 pmoles of dsODN. Seven days post transfection, genomic DNA was isolated from the different samples using the Quick-DNA™ Miniprep Plus Kit (Zymo Research Cat#D4069). Genomic DNA samples were quantified via Qubit and normalized to 50 ng/ul. Genomic DNA was fragmented via enzymatic digestion using fragmentase (NEB); 500 ng of gDNA was digested with 2 ul of the enzyme in a total volume of 20 μL at 37 C for 18 min in a thermocycler. The fragmented DNA was then purified with AMPure XP beads at a ratio of 1:1. A total of 500 ng of fragmented DNA was treated with the NEBNext^®^ Ultra™ II End Repair/dA-Tailing Module following user manual instructions, followed by ligation with the Illumina sequencing adapters using the NEBNext^®^ Ultra™ II Ligation Master Mix. The next step was to perform two rounds of nested anchored PCR with primers complementary to the oligo tag for target enrichment. Libraries were analyzed using a fragment analyzer, quantified via Qubit, and sequenced using MiSeq. Data processing and analysis were carried out using the GUIDE-seq analysis pipeline (Guideseq, 2024). The GUIDE-seq dataset was uploaded and published in Zenodo (Creators Morell, 2024).

### Statistical analysis and software

All data analyses were performed with Prism 10.0.2 (GraphPad, Boston, MA) and Excel 16.8.2 (Microsoft, Redmond, WA). Data were compared by ANOVA with the recommended post-test. Plasmid sequences were designed in Benchling (San Francisco, CA). FlowJo 10.9.0 (Treestar, OR) was used to analyze the fcs files exported from SpectroFlo and Attune NxT software. Representative flow plots were exported from FlowJo. Figures were created and organized using Illustrator 28.0 (Adobe, San Jose, CA). A *p*-value of less than 0.05 was defined as statistically significant.

## Results

### Manufacturing of non-viral, TRAC-B2M-PD1 triple-knockout GD2 CAR T cells

**FIGURE 1:**
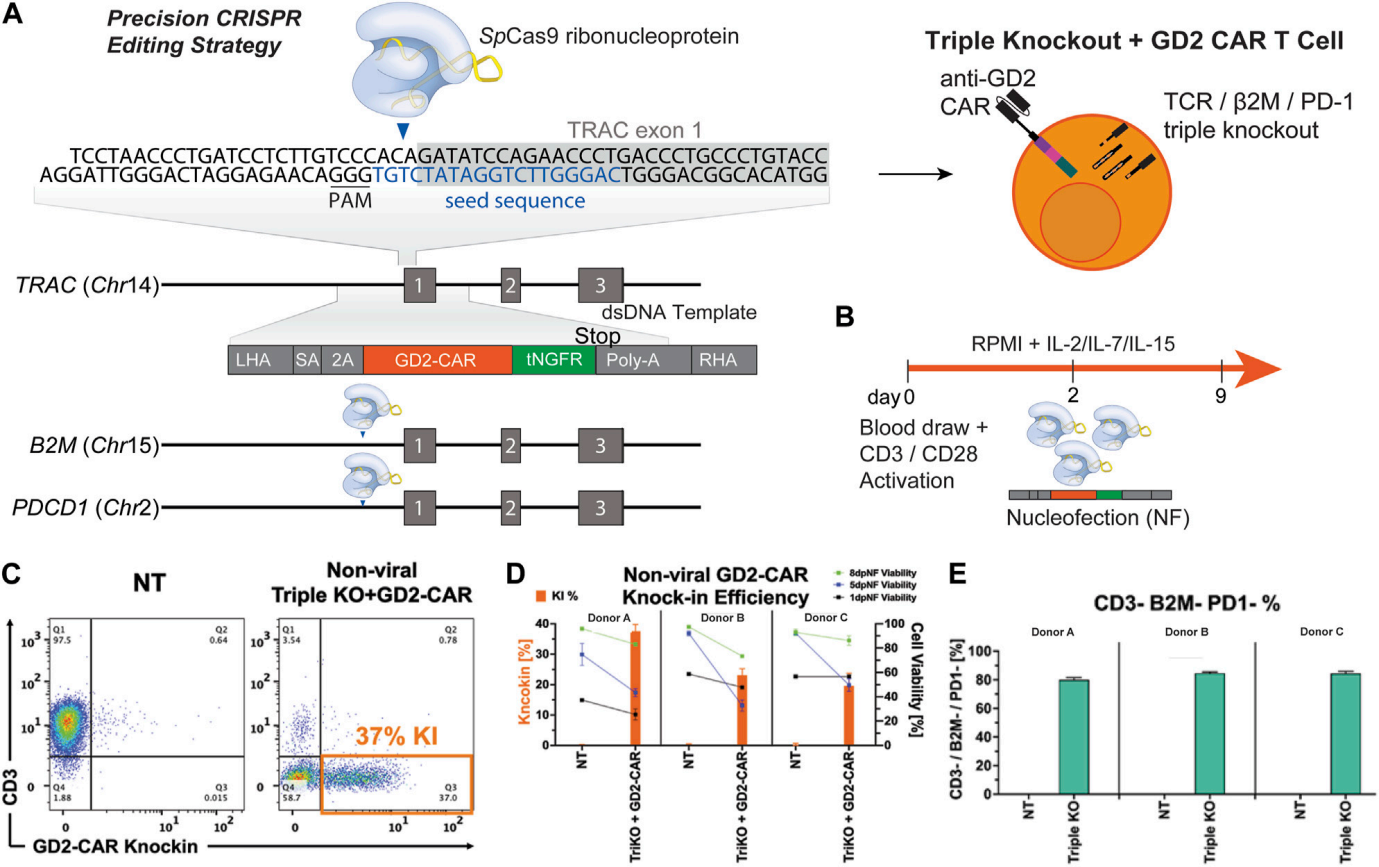
Multiplex gene editing to manufacture triple-knockout (CD3^−^/β2M^−^/PD-1^−^) anti-GD2 CAR T cells. **(A)** Schematic of a CAR construct consisting of 500 bp left and right homology arms, third-generation anti-GD2 CAR, and a tNGFR tag inserted into the *TRAC* locus with simultaneous knockout of the *B2M* and *PDCD1* genes. **(B)** Schematic of GD2 CAR T-cell manufacturing. **(C)** Representative flow plots depicting the expression of CAR versus CD3 for triple-knockout CAR T cells and non-transduced (NT) T cells. **(D)** Bar graphs comparing the CAR knock-in rate and viability on days 1, 5, and 8 post-nucleofection of NT and TRAC-B2M-PD1 triple-knockout GD2-CAR T cells across three donors. **(E)** Bar graphs depicting the percentage of T cells negative for CD3, β2M, and PD-1 across three donors. CAR, chimeric antigen receptor; tNGFR, truncated nerve growth factor receptor; RNP, ribonucleoprotein.

We designed multiplex-edited CAR T cells by targeting single-guide RNAs (sgRNA) to *a*) the TCR alpha chain (*TRAC*), limiting GVHD by allogeneic CAR T cells; *b*) beta microglobulin (*B2M*), inducing loss of HLA class 1 to avoid T cell-mediated rejection of allogeneic CAR T cells; and *c*) programmed cell death protein 1 (*PD1*), to prevent exhaustion and improve T-cell fitness. We integrated a homology-directed repair (HDR) donor template containing a third-generation anti-GD2 CAR transgene (Mueller et al., 2022) and a tNGFR tag flanked by homology arms into the *TRAC* locus (Figure 1A). Human primary T cells were isolated from healthy donors and activated for 2–3 days, after which they were nucleofected with RNP’s knocking out TRAC, β2M, and PD1 and knocking-in the dsDNA CAR donor template. TRAC-B2M-PD1 triple-knockout GD2 CAR T cells were then expanded for 7 more days in IL-7 and IL-15 and then cryopreserved for future analysis (Figure 1B). We assessed CAR knock-in efficiency on day 5 post-nucleofection of manufacturing and saw that 20%–40% of T-cells successfully integrated the CAR construct across three donors. Immediately post-nucleofection, T-cell viability did not exceed 60%, but it improved as cells expanded in IL-7/IL-15, with notably lower survival in gene-edited cells compared to non-transfected controls (Figures 1C and D). Over 80% of T cells remained triple-negative (CD3^-^/β2M^−^/PD-1^-^) when stimulated with PMA/ionomycin (Figure 1E).

### Low translocation rate and off-target editing in TRAC-B2M-PD1 triple-knockout T cells

**FIGURE 2:**
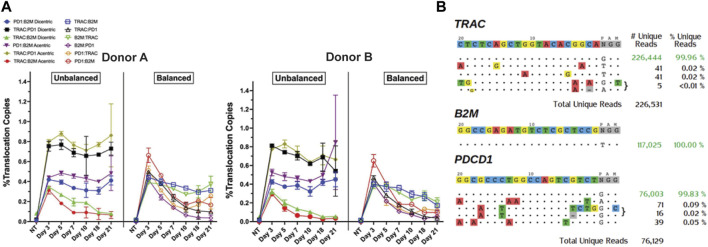
Translocation formation and off-target editing in multiplex-edited T-cell products. **(A)** Chromosome abnormalities were investigated with ddPCR for the time-course quantification of unbalanced and balanced translocations. Line plots depict the percentage of translocation-positive molecules in non-transduced and TRAC-B2M-PD1 triple-knockout T-cells on days 3, 5, 7, 10, 18, and 21 post-nucleofection for two separate healthy donors (HD-A and HD-B). **(B)** Off-target analysis for each guide RNA was assessed using GUIDE-seq. Greater than 99% of reads for double-strand break formation for all three guides mapped to the intended on-target site.

Simultaneous multiplex editing of T-cells can introduce chromosomal abnormalities, such as translocations and off-target editing (Poirot et al., 2015; Qasim et al., 2017; Benjamin et al., 2020; Stadtmauer et al., 2020; Sasu et al., 2023). To assess the frequency of these events after multiplex gene deletions, we manufactured triple-knockout T cells without knock-in of the GD2-CAR, and we employed ddPCR to analyze the translocation events and GUIDE-seq to investigate off-target editing. T cells from two independent donors (HD-A and HD-B) were analyzed by ddPCR 3 days post-nucleofection (dpNF) up to 21 days from manufacturing. Both balanced and unbalanced translocations were observed in less than 1% of all cells (Figure 2A). The percentage of unbalanced TRAC:B2M and all balanced translocations peaked at day 3 dpNF and decreased at later time points, reaching a significantly lower threshold at 21 dpNF (Figure 2A). The percentage of unbalanced TRAC:PD1 and PD1:B2M translocations remained constant up to day 21, indicating an increased unequal exchange of genetic material at these loci (Figure 2A). Using GUIDE-seq, sgRNA specific for *TRAC* and PD1 demonstrated only three off-target sites, with no off-target sites detected for B2M sgRNA (Figure 2B). These data support that the idea that multiplexed T-cell editing with sgRNAs demonstrates high efficacy and fidelity in more than 99% of T cells.

### Favorable memory phenotypes in TRAC-B2M-PD1 triple-knockout GD2 CAR T cells

**FIGURE 3:**
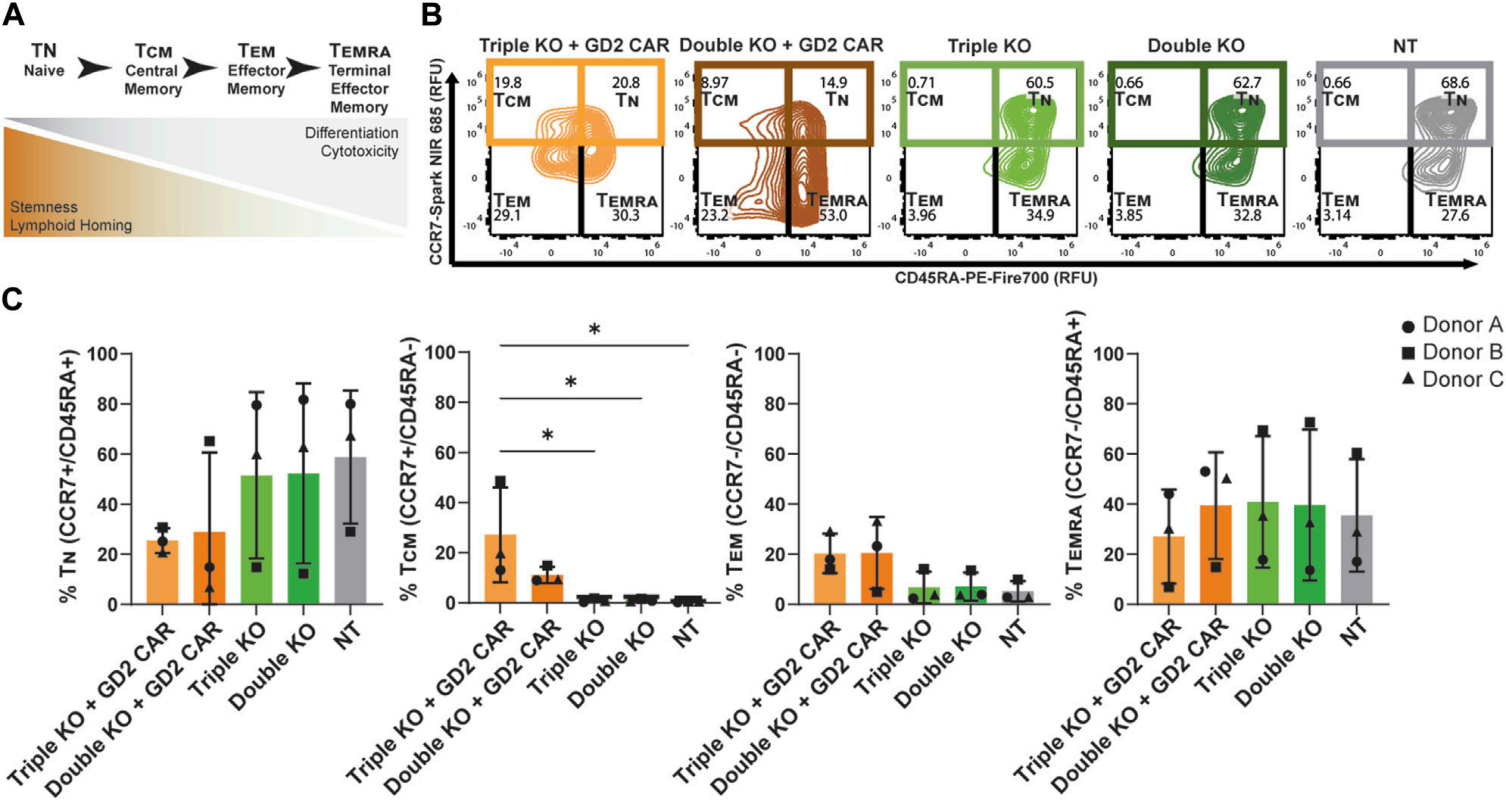
TRAC-B2M-PD1 triple-knockout GD2 CAR T-cell products are enriched for central memory phenotypes. **(A)** Schematic of the definition of T-cell phenotypes: T_N_ (naïve, CD45RA^+^/CCR7^+^), T_CM_ (central memory, CD45RA^−^/CCR7^+^), T_EM_ (effector memory, CD45RA^−^/CCR7^-^), and T_EMRA_ (terminal effector memory, CD45RA^+^/CCR7^-^), as defined by their inherent properties. **(B)** Representative contour plots of CCR7 vs. CD45RA expression depicting the relative percent of T_N_, T_CM_, T_EM_, and T_EMRA_ in the thawed pre-infusion product of TRAC-B2M-PD1 triple-knockout (KO) GD2 CAR T cells (CAR^+^, TCR^−^, β2M^−^, PD-1^-^), TRAC-B2M double-KO CAR T cells (CAR^+^, TCR^−^, β2M^−^, PD-1^+^), TRAC-B2M-PD1 triple-KO T cells (CAR^−^, TCR^−^, β2M^−^, PD-1^-^), TRAC-B2M double-KO T cells (CAR^−^, TCR^−^, β2M^−^, PD-1^+^), and non-transfected T cells (CAR^−^, TCR^+^, β2M^+^, PD-1^+^). **(C)** Bar graph of the relative percentage of T_N_, T_CM_, T_EM_, or T_EMRA_ in each population. Three donors. Error bars represent the standard deviation. Statistical significance was determined with Brown–Forsythe and Welch ANOVA tests using Dunnett’s T3 test for multiple comparisons; *p < 0.05.

Higher amounts of naive and central memory T-cells in pre-infusion CAR T products have been correlated with increased persistence and potency post-infusion *in vivo* (Gattinoni et al., 2011; Biasco et al., 2021). This phenotype can be characterized by the expression of surface markers like CD45RA and CCR7, where naïve T-cells (T_N_) are CD45RA^+^/CCR7^+^, central memory T-cells are CD45RA^−^/CCR7^+^, effector memory T-cells (T_EM_) are CD45RA^−^/CCR7^-^, and terminal effector memory T-cells (T_EMRA_) are CD45RA^+^/CCR7^-^ (Figure 3A) (Geginat et al., 2003; Shen et al., 2022). The expression levels of these memory markers were found using flow cytometry, distinguishing populations based on CD45, CAR, and TCR expression in thawed T-cell products sorted by FACS (Supplementary Figure S1). The CD45^+^/CAR^+^/TCR^−^ populations of TRAC-B2M-PD1 triple- and TRAC-B2M double-knockout GD2 CAR T cells, CD45^+^/CAR^−^/TCR^−^ populations of TRAC-B2M-PD1 triple- and TRAC-B2M double-knockout T cells, and CD45^+^/CAR^−^/TCR^+^ populations of non-transfected T cells were profiled. Over 50% of TRAC-B2M-PD1 triple-knockout GD2 CAR T cells had a naïve or central memory phenotype (Figure 3B). TRAC-B2M-PD1 triple-knockout T cells, TRAC-B2M double-knockout T cells, and non-transfected T cells had a higher magnitude of naïve T-cells than those with a GD2 CAR knock-in, and TRAC-B2M-PD1 triple- and TRAC-B2M double-knockout T cells had significantly lower central memory populations than TRAC-B2M-PD1 triple-knockout GD2 CAR T cells (Figure 3C). These data suggest that the knock-in of a CAR transgene coupled with a TRAC-B2M-PD1 triple-knockout could enrich central memory phenotypes. No significant differences were observed in CD8 and CD4 expression among T cells, with over 60% of TRAC-B2M-PD1 triple-knockout CAR T cells being CD8^+^ (Supplementary Figure S2).

### High *in vitro* potency of TRAC-B2M-PD1 triple-knockout GD2 CAR T cells

**FIGURE 4:**
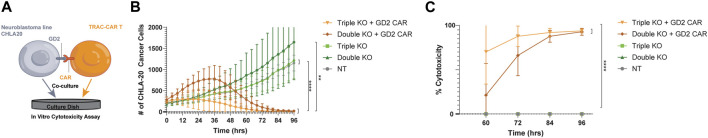
Triple-knockout GD2-CAR T cells are potent against GD2^+^ neuroblastoma targets. **(A)** GD2^+^ neuroblastoma (CHLA-20) cells were seeded onto 96-well plates for 24 h before the addition of cryopreserved T cells. Thawed TRAC-B2M-PD1 triple-knockout (KO) + GD2 GD2 CAR T cells (CAR^+^, TCR^−^, β2M^−^, and PD-1^-^), TRAC-B2M double-KO GD2 CAR T cells (CAR^+^, TCR^−^, β2M^−^, and PD-1^+^), TRAC-B2M-PD1 triple-KO T cells (CAR^−^, TCR^−^, β2M^−^, and PD-1^-^), TRAC-B2M double-KO T cells (CAR^−^, TCR^−^, β2M^−^, and PD-1^+^), or non-transfected T cells (CAR^−^, TCR^+^, β2M^+^, and PD-1^+^) were added at a 1:1 effector:target (E:T) ratio and followed by live cell imaging. GFP fluorescence (CHLA-20 viability) was measured continuously over 96 h and graphed over time. **(B)** Change in GFP count (cell number) and **(C)** percent CHLA-20 cancer cells lysed after co-culture with TRAC-B2M-PD1 triple-KO GD2 CAR T cells, TRAC-B2M double-KO GD2 CAR T cells, TRAC-B2M-PD1 triple-KO T cells, TRAC-B2M double-KO T cells, and NT T cells at a 1:1 E:T ratio. **(C)** Percent cytotoxicity of GFP, green fluorescent protein. Three donors. Error bars represent the standard deviation. Statistical significance was determined with Brown–Forsythe and Welch ANOVA tests using Dunnett’s T3 test for multiple comparisons; *p < 0.05; **p < 0.01.

To investigate the potency of triple-knockout GD2-CAR T cells, we measured the cytotoxicity after co-culture with the GD2^+^ neuroblastoma cell line, CHLA-20. CHLA-20 target cells were seeded in 96-well plates and grown for 24 h, during which thawed GD2 CAR T cells sorted by FACS with triple-knockout (CD3^-^/β2M^−^/PD-1^-^) were compared as effectors to double-knockout (CD3^-^/β2M^−^) primary T cells or non-transfected T cells manufactured from three healthy donors. Thawed T-cell products were added at a 1:1 effector:target (E:T) ratio after 24 h (Figure 4A). TRAC-B2M-PD1 triple- and TRAC-B2M double-knockout GD2 CAR T cells lysed tumor targets at a similar efficacy, while non-CAR transduced, TRAC-B2M-PD1 triple-, and TRAC-B2M double-knockout T cells showed no cytotoxicity, indicating the need for antigen specificity by the CAR and the inability for allogeneic cytotoxicity due to a lack of a TCR (Figure 4B). The TRAC-B2M-PD1 triple-knockout GD2 CAR T cells were the only group that showed over 70% and 85% cytotoxicity at 60 and 72 h, respectively (Figure 4C), suggesting that the knockout of PD-1 may increase the potency for GD2 CAR T cells against neuroblastoma.

## Discussion

This study used multiplex editing with CRISPR/Cas9 to manufacture, for the first time, GD2 CAR T cells that lacked the expression of the endogenous TCR, β2M, and PD-1 as a potential allogeneic “off-the-shelf” therapy. This approach led to minimal chromosomal abnormalities and off-target editing, showing feasibility and safety. To assess the potency, high cytotoxicity against GD2^+^ human neuroblastoma cells was observed *in vitro*, and high proportions of central memory T-cells were also observed.

To prevent inhibitory effects and ameliorate the exhausted phenotypes (Araki et al., 2013; Park et al., 2016), PD-1 has been disrupted in CAR T cells to improve anti-tumor efficacy and persistence (Rupp et al., 2017; Guo et al., 2018; Hu B. et al., 2019; Choi et al., 2019; Dai et al., 2019; Magnani et al., 2020; McGowan et al., 2020; Wang et al., 2021; Khan and Sarkar, 2022). Additional edits targeting the *TRAC* or *β2M* loci to generate universal, allogeneic CAR T cells have succeeded in generating highly-edited T cells resistant to host rejection with demonstrated potency against tumors (Ren et al., 2017; Dai et al., 2019; Magnani et al., 2020). Multiplexed editing has typically used CRISPR/Cas9 to knock out genes of interest, but it also frequently uses lentiviral vectors for transgene knock-in (Kebriaei et al., 2016; Ren et al., 2017; Magnani et al., 2020). Viral vector production can be a barrier to scaling up from laboratory production, given the cost and long lead time needed (Ran et al., 2020). Non-viral gene delivery vectors can potentially shorten lead times and complexity in manufacturing to overcome those barriers, although at lower knock-in efficiency than viral vectors (Foy et al., 2022; Kath et al., 2022; Madison et al., 2022; Ye et al., 2022; Webber et al., 2023). Recent approaches have electroporated CRISPR/Cas9 and HDR templates into T cells to non-virally deliver the CAR transgene into the *TRAC* locus under the control of the endogenous promoter, and they have demonstrated on-target editing and increased fractions of naïve or stem-cell memory T cells (Eyquem et al., 2017; Schober et al., 2019; Mueller et al., 2022). This study builds upon the production of TRAC-GD2 CAR T cells manufactured in this way (Sasu et al., 2023), but it introduces additional edits at *β2M* and *PDCD1* loci to produce a non-viral, allogeneic, and potentially exhaustion-resistant CAR T-cell product.

Whenever multiple DNA double-strand breaks are generated within cells, the formation of chromosomal translocations is possible (Bishop et al., 2021). Furthermore, off-target editing for each Cas9 RNP used can be additive. Simultaneous *TRAC* and CD52 disruption by TALENs in CD19 CAR T cells and CRISPR/Cas9-manufactured T cells expressing a TCR caused karyotypic anomalies in approximately 5% of cells, suggesting a moderate rate of translocation (Poirot et al., 2015; Qasim et al., 2017; Benjamin et al., 2020; Stadtmauer et al., 2020). Using a ddPCR assay at multiple points during the manufacturing of TRAC-B2M-PD1 triple-knockout GD2 CAR T cells, RNPs with the chosen guide RNAs universally produced translocations in less than 1% of all cells. There were more unbalanced than balanced translocations, indicating an unequal exchange of genetic information in those cells. This observation was most prominent between the TRAC:PD-1 and PD-1:B2M loci, respectively. Investigating the edits at these sites for translocations would, therefore, be imperative to ensure the safety of a clinical product. Each of the guide RNAs had <0.5% off-target effects by GUIDE-seq, demonstrating high-fidelity and on-target editing of this multiplex editing manufacturing process. However, future studies should require additional sequencing of disrupted gene loci to be certain of minimal off-target insertion of the donor template and to characterize the extent of biallelic or monoallelic editing for both knock-in and knockout. Our screening with ddPCR and GUIDE-seq can be used for other CAR T products with CRISPR-Cas9-mediated gene disruptions to further improve the fidelity of guide RNAs and prevent translocation formation.

The non-viral, multiplex editing process in this study can be adapted to target other loci and editing strategies to improve adoptive T-cell therapies. For example, there have been efforts to use base editing instead of CRISPR to ablate the endogenous TCR and CD7 to limit fratricide between T cells, which showed reduced levels of translocations (Georgiadis et al., 2021). Applying this strategy to our triple-knockout GD2 CAR T cells could potentially further reduce the translocation rate below 0.9%. Additionally, *TRAC-*CAR T cells have been shown to have improved stem cell memory profiles (Nakazawa et al., 2020; Sasu et al., 2023), and the TRAC-B2M-PD1 triple-knockout CAR T cells have memory phenotypes consistent with single *TRAC* knockout CAR cells. Efforts to optimize cytokines supplemented in expansion media (Xu et al., 2014; Cappabianca et al., 2024; Pham et al., 2024), *ex vivo* metabolic engineering (Amini and Veraitch, 2019; Shen et al., 2022; Ye et al., 2022), and rapid manufacturing of T cells (Ghassemi et al., 2022) have all been shown to increase the stem cell memory populations of CAR T cells. These complementary strategies may be able to further increase the potency of TRAC-B2M-PD1 triple KO CAR T cells against tumors and improve persistence post-infusion (Gattinoni et al., 2011; Biasco et al., 2021). Our study can serve as a platform for future studies to test for improved *in vivo* potency in multiple GD2-expressing indications, like neuroblastoma, Ewing’s sarcoma, and non-small-cell lung cancers. Finally, the simultaneous disruption of the *TRAC* and *B2M* loci has been demonstrated to reduce GVHD and rejection by the host as a means of producing universal allogeneic CAR T cells (MacLeod et al., 2017; Martínez Bedoya et al., 2021). The manufacturing of allogeneic cell therapies will require that large batches be cryopreserved pre-infusion to be thawed as an off-the-shelf product, but there is the risk that cryopreservation changes the phenotype of the fresh cells and/or impairs potency. Cryopreserved TRAC-B2M-PD1 triple-knockout GD2 CAR T cells maintain a memory phenotype and potency post-thaw, which is a particularly important step toward translation to allogeneic manufacturing.

## Data Availability

The original contributions presented in the study are included in the article/Supplementary Material; further inquiries can be directed to the corresponding authors.
