# Supplementary material for: Non-viral expression of chimeric antigen receptors with multiplex gene editing in primary T cells
Source: Front Bioeng Biotechnol. 2024 May 31;12:1379900. doi: 10.3389/fbioe.2024.1379900 (PMC11177325; doi:10.3389/fbioe.2024.1379900)
Supplement: Supplementary file 1 [file DataSheet1.PDF]

Supplementary Material

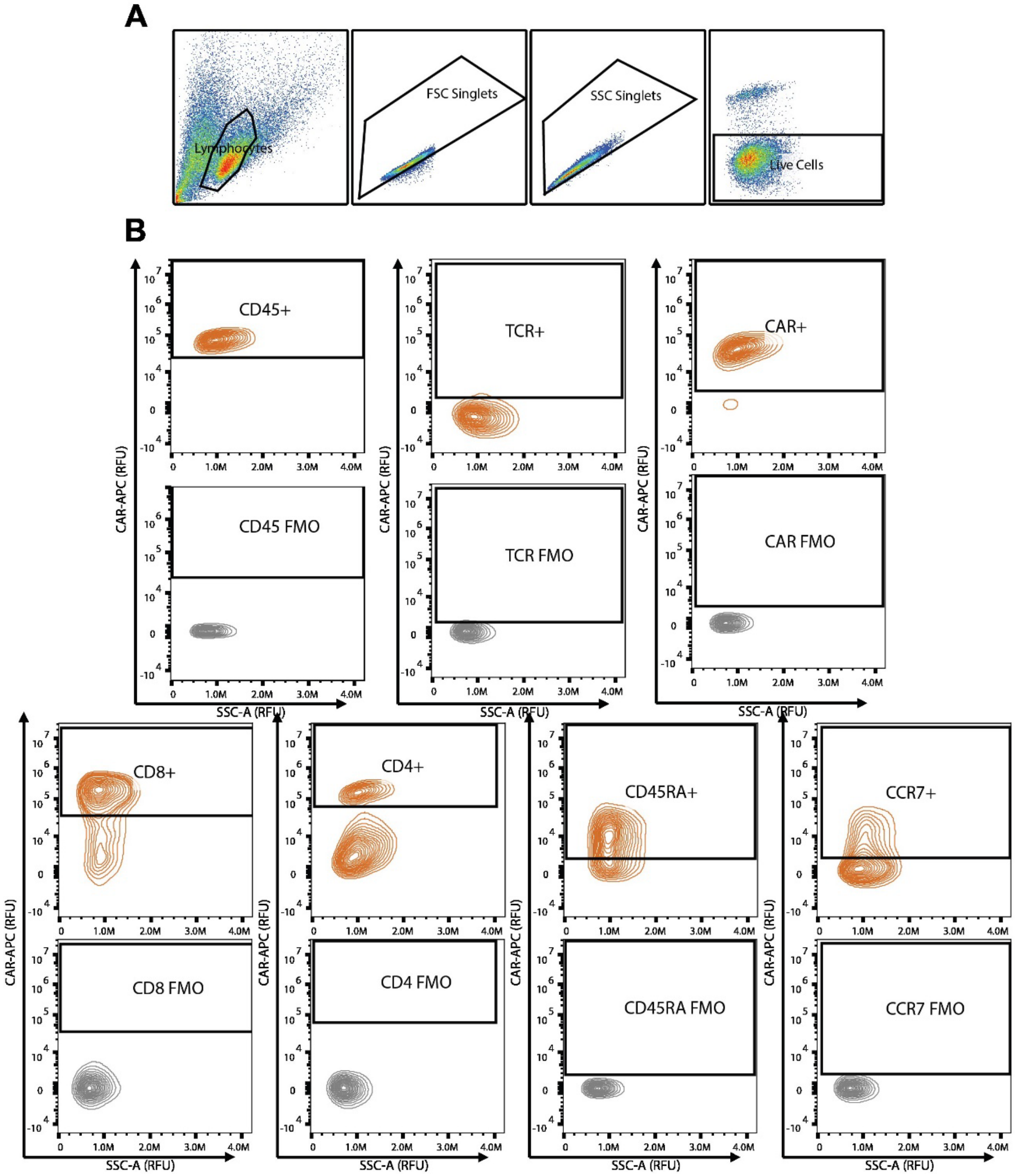

Figure S1. Post-Manufacturing CAR T Cell Gating Strategy. (A) Gating strategy for analysis of spectral immunophenotyping flow cytometry data of CAR T cells post-manufacturing. **(B)** FMO's and representative positive populations depicting positive and negative gates.

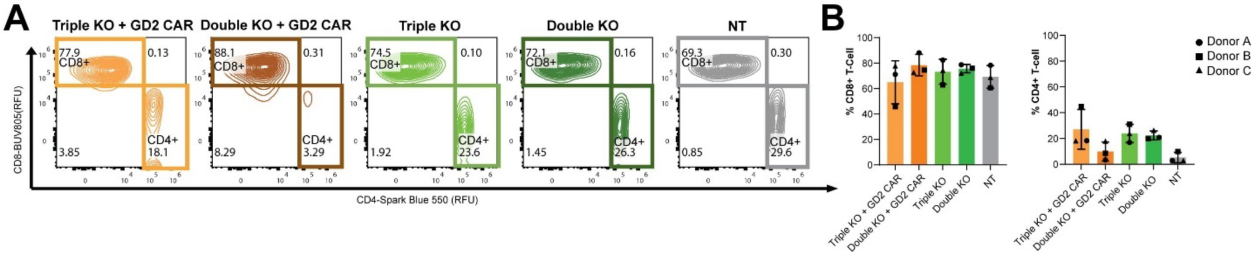

Figure S2: Triple knockout GD2-CAR T cell expression of CD8 and CD4. (A) Contour plots of the CD8 and CD4 expression in each population of triple knockout (KO) + GD2 CAR T cells (CAR^+, TCR^-, $\beta 2M^-$, $PD\text{-}1^-$), double KO CAR T cells (CAR^+, TCR^-, $\beta 2M^-$, $PD\text{-}1^+$), triple KO T-cells (CAR^-, TCR^-, $\beta 2M^-$, $PD\text{-}1^-$), double KO T-cells (CAR^-, TCR^-, $\beta 2M^-$, $PD\text{-}1^+$), and non-transduced (NT) T-cells (CAR^+, TCR^+, $\beta 2M^+$, $PD\text{-}1^+$). **(B)** Bar graphs depicting the percentage of $CD8^+$ and $CD4^+$ T-cells in each population.
